# Roles of Keber's valve and foot chamber for foot manipulation in the mussel *Nodularia douglasiae*

**DOI:** 10.1242/bio.039859

**Published:** 2018-12-10

**Authors:** Eriko Seo, Yoshiteru Seo

**Affiliations:** 1 Department of Marine Ecosystem Dynamics, Division of Marine Life Science, Atmosphere and Ocean Research Institute, The University of Tokyo, Kashiwa, 277-8564, Japan; 2 Department of Regulatory Physiology, Dokkyo Medical University School of Medicine, Tochigi, 321-0293, Japan

**Keywords:** Keber's valve, Heartbeat, Burrowing, Unionoidae, Magnetic resonance imaging

## Abstract

In order to analyse the roles of Keber's valve for foot manipulation in the mussel *Nodularia douglasiae*, the anatomy and haemolymph flow in the cardiovascular system were detected by magnetic resonance imaging. The superficial layer of the foot was covered by a dense muscle layer, which extended to the dorsal side and connected with the shell. This closed space, the foot chamber, had an inlet (anterior aorta) and an outlet (Keber's valve). At rest, in the beginning of the systolic phase, flows in the anterior aorta and the pedal artery increased, followed by the pedal and visceral sinuses. Then these flows ceased at the end of the systolic phase, followed by inflow to the ventricle in the diastolic phase; therefore, the compliance of the foot chamber is low enough to transfer pressure pulses to the visceral sinus. Extension of the foot started with relaxation of the foot muscle, so the compliance of the foot chamber increased. Then, Keber's valve closed so that the haemolymph filled the foot haemocoel. Retraction of the foot is initiated by the opening of Keber's valve. Judging from these results Keber's valve and the foot chamber are essential for circulation at rest, foot extension and retraction.

This article has an associated First Person interview with the first author of the paper.

## Abstract (precis)

**Summary:** Opening and closing of Keber's valve in the mussel was visualized using MRI. Keber's valve and the foot chamber are essential for circulation at rest and during foot extension and retraction.

## INTRODUCTION

The main functions of the circulation system are (1) convection of solutes and heat, and (2) transmission of forces (Schmidt-Nielsen, 1983). The Mollusca Bivalvia has an open circulatory system and hydrostatic pressure is considered to be the major driving force of motion of the body (Brand, 1972; Trueman, 1983). Burrowing activity has been studied for many years (Morton, 1964; Trueman, 1983; Trueman et al., 1986; Winter et al., 2012). The burrowing process involves the extension and probing of the foot into the sand, followed by adduction of the shell and retraction of the foot, drawing the shell down towards the anchored tip of the foot (Brand, 1972). Brand (1972, 1976) observed pressures in the ventricle and the interstitial space of the foot (pedal haemocoel) of the *Anodonta anatine*. In the foot haemolymph is supplied by the pedal artery, filling the pedal haemocoel, and then it is accumulated into the pedal sinus. At the proximal end of the pedal sinus, Keber's valve controls venous return to the heart via the visceral sinus (Brand, 1972; Keber, 1851). During the extension of the foot, the pressure of the haemocoel increased gradually followed by an increase of the systolic ventricular pressure up to 50 mm H_2_O. Then, at the adduction of the shell, the pressure of the haemocoel was transiently increased to several times that of the ventricular pressure (Brand, 1972). This high pressure might be caused by the contraction of the adductor and retractor muscles, and it is necessary to keep the Keber's valve closed. Therefore, control of the opening and closing of Keber's valve is essential for the burrowing process. However, as far as we have found in the related literature, no one has observed the opening and closing of Keber's valve during the foot extension or retraction. In addition, it has also been postulated that the outflow of haemolymph is allowed only via Keber's valve (Trueman, 1966). However that is not likely the case, because the foot contains not only the haemocoel, but also the intestine, digestive glands and gonad cells. The proximal end of the foot is not sealed by the dense muscle layer, and the haemolymph could leak into the dorsal visceral mass including the stomach, heart and gills. Thus, it may be that the pressure of the pedal haemocoel could not be retained during the foot extension. Therefore, in this study, we first observed the anatomical structure of the foot to confirm the route of the outflow of the haemolymph. Then, we measured the haemolymph flow (hereafter, ‘flow’ alone) in the ventricle, pedal artery, pedal sinus, visceral sinus and Keber's valve in the resting state, as well as during foot extension and retraction, in order to test the conventional model of extension and retraction of the foot.

*Nodularia douglasiae* (Gray, 1833) was selected as the experimental animal, since this is in the same family of Unionoidae, *A. anatine* (Linnaeus, 1758). Unfortunately, *N. douglasiae* was not the Unionoidae in which the presence of the Keber's valve was previously described (Keber, 1851; Schwanecke, 1913; Willem and Minne, 1898, Brand, 1972), so we conducted a histological study on Keber's valve and the adjacent sinuses and organs in *N. douglasiae*. The flow and heartbeats were detected by magnetic resonance imaging (MRI), which was employed to detect the flow of seawater in the mantle cavity and to measure the heart rate as in our previous reports (Seo et al., 2014b, 2016). Since we could not force the mussels to rest, extend or retract the foot, we conducted more than 100 sessions of MRI measurements. Then the images obtained were sorted into the resting, extension and retraction conditions of the foot. The interbeat interval (IBI) of the heart, as well as the flow in the anterior aorta, pedal artery, pedal sinus and the visceral sinus, obtained under the three conditions mentioned above, were examined to clarify the roles of Keber's valve in the extension and retraction of the foot.

## RESULTS

### The structure of Keber's valve, the dorsoventral muscle and the foot

**Fig. 1.**
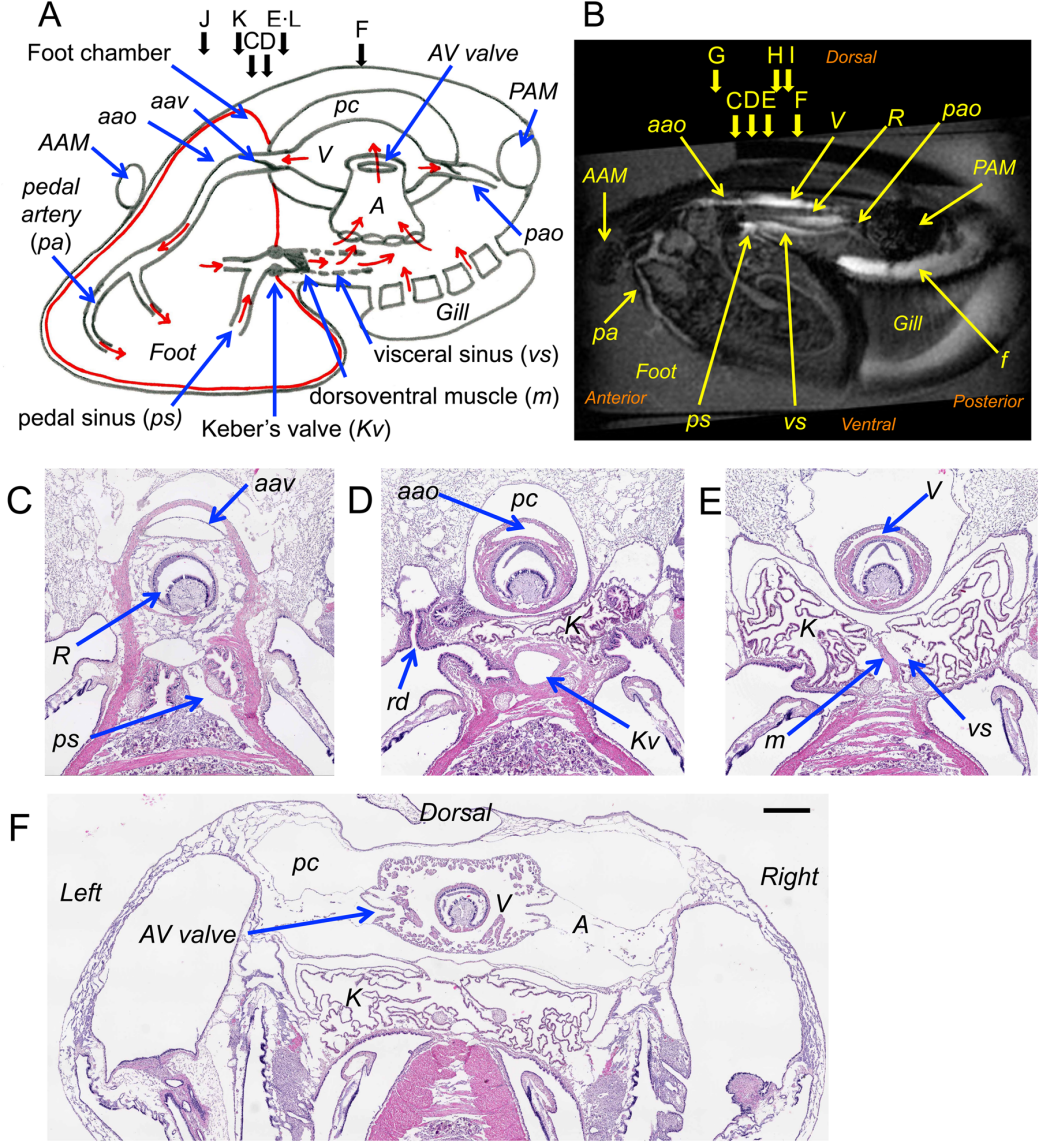

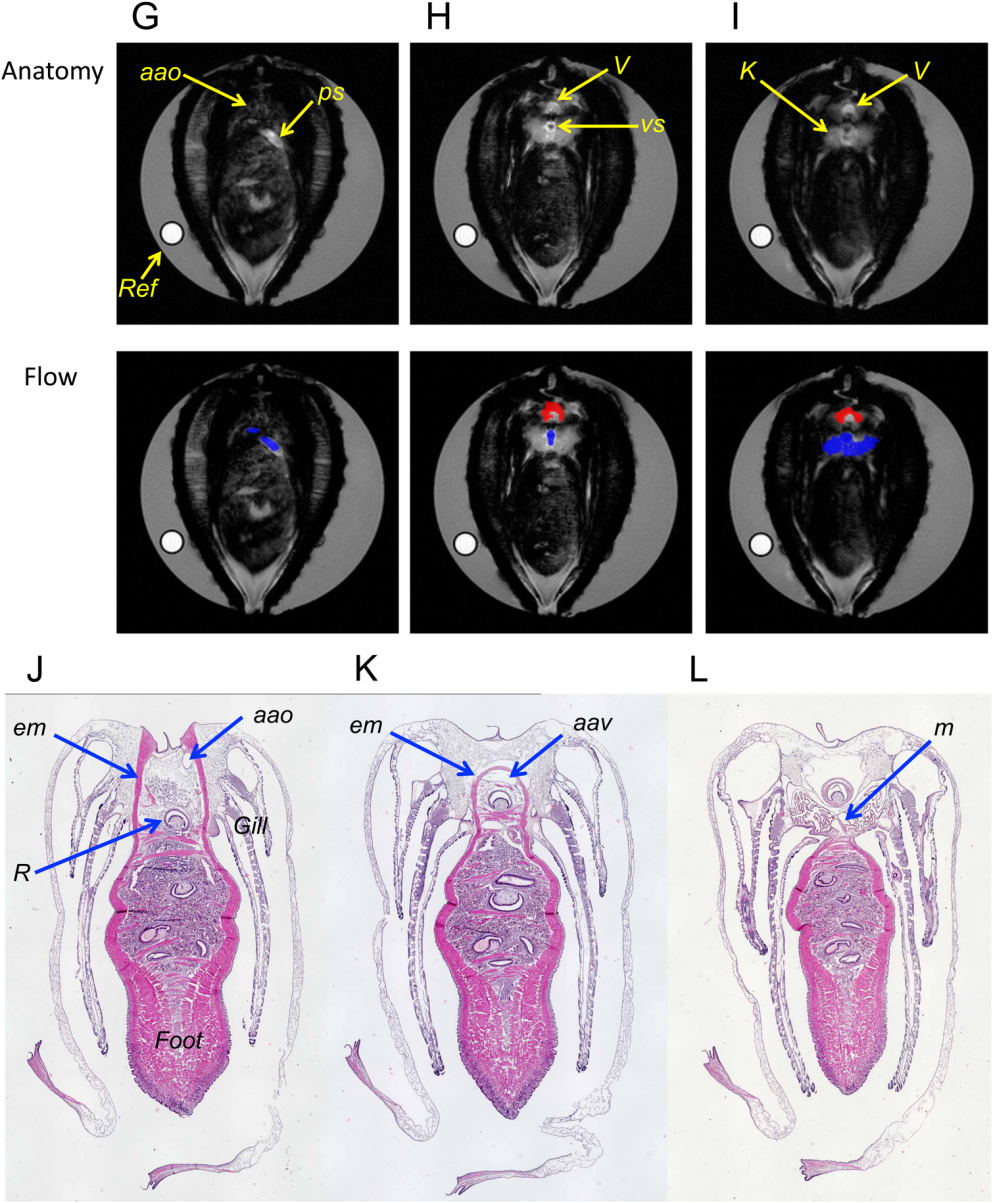
**Cardiovascular system of *N.*** ***douglasiae.*** (A,B) Summary of anatomical structure. (A) Schematic diagram of the heart and adjunct vessels in the longitudinal view. The direction of the haemolymph stream is shown with red arrows. The red bold line indicates the wall of the foot chamber. For clarity, the digestive organs and kidney have been omitted. (B) Mid-longitudinal T_1w_-MR image of a living mussel. The position of the section is indicated by arrows labelled C to L. (C–F) Transverse section of the PFA fixed mussel. Scale bar: 500 µm. (C) Section at the level of the pedal sinus (*ps*) and the anterior aortic valve (*aav*). (D) Section at the Keber's valve (*Kv*) level. (E) Section at the level of the dorsoventral muscle (*m*) and the visceral sinus (*vs*). (F) Section at the level of the auriculoventricular valves (*AV* valve). Also refer to Movie 1. (G–I) Flow of haemolymph in the living mussel detected by phase-contrast MRI. Anterior- and posterior-directed flow of water are shown in red and blue, respectively. (G,H,I) Sections at 5 mm, 2 mm and 1 mm anterior from the AV valve. (J–L) The foot chamber. Transverse section of the PFA fixed mussel showing the superficial muscle layer of the foot. (J) Section at the posterior side of the stomach. The muscle layer (*em*) extends to the dorsal side of the shell. (K) Section at the level of the anterior aortic valve (*aav*). The muscle layer (*em*) covers the anterior aorta. (L) Section at the level of the dorsoventral muscle (*m*). The muscle layer of the foot covers only the foot. Labelled features: *A*, auricle; *AAM*, anterior adductor muscle; *aao*, anterior aorta; *aav*, anterior aortic valve; *AV* valve, auriculoventricular valves; *em*, muscle layer; *f*, water stream in the upper mantle cavity underneath the *PAM*; *K*, kidney; *Kv*, Keber's valve; *m*, dorsoventral muscle; *pa*, pedal artery; *PAM*, posterior adductor muscle; *pao*, posterior aorta; *pc*, pericardial cavity; *ps*, pedal sinus; *R*, rectum; *rd*, renal duct; *Ref*, reference capillary containing 0.5 mM MnCl_2_; *V*, ventricle; *vs*, visceral sinus.

The cardiovascular system of the *N. douglasiae* was analysed by using high-resolution three-dimensional (3D) MR images and light microscopic images of haematoxylin-eosin (HE) staining, and the important anatomy elements were summarised as a schema (Fig. 1A) and a mid-sagittal image of T_1_-weighted gradient-echo imaging (T_1w_-MRI) (Fig. 1B). The heart consists of a ventricle and pair of auricles in the pericardial cavity. The anterior aorta supplies haemolymph to the foot and digestive organs. The posterior aorta supplies haemolymph to the mantles. Both of the aortae have a single-flap valve to prevent backflow to the ventricle (Fig. 1C). The cross-sectional area of the anterior aorta distal side of the valve (1.1×10^5^ µm^2^) was nine times bigger than that of the posterior aorta (1.2×10^4^ µm^2^). Haemolymph in the foot accumulates in the pedal sinus (Fig. 1C), then it passes through Keber's valve (Fig. 1D) to the visceral sinus (Fig. 1E). After being perfused in the interstitial space of the kidney, the haemolymph returned to the auricula (Fig. 1F). The auriculoventricular (AV) valve is a slit-shaped valve (1 mm in length), and it is a dual-flap valve with dorsal and ventral flaps to prevent backflow to the auricula (Fig. 1F). Keber's valve was positioned at the anterior end of the pericardial cavity near the renal ducts (Fig. 1D). The shape is similar to a sphincter muscle with a diameter of 0.5 mm and a thickness of 0.2 mm. The dorsoventral muscle is a thin, flat, trapeziform muscle connected between the dorsal and the ventral walls of the visceral sinus (Fig. 1E). Please consult Movie 1 showing HE images of Keber's valve to the dorsoventral muscle at a 50 µm interval.

The direction of the haemolymph flow was detected by phase-contrast gradient-echo sequences (PC-MRI) in a range of 7.5 mm s^−1^. Typical results in the anterior-posterior direction were shown in Fig. 1G–I. Anterior directed flow in the ventricle was detected, and posterior directed flow in the visceral sinus and pedal sinus were also detected. As expected previously, anterior directed flow of the anterior aorta and posterior directed flow of the posterior aorta were also detected (data not shown) (Brand, 1972). Flow in the kidney and interstitial space of the kidney were detected by T_1w_-MRI as a high signal intensity. Posterior flow in the kidney was detected in Fig. 1I, but could not be detected in Fig. 1H because the velocity was lower than the lower limit of detection. The flow of the anterior aorta in the aortic valve was also hard to detect because of a mixture of the anterior and posterior flow of haemolymph (Fig. 1G). Judging from these results, the direction of the stream was shown in Fig. 1A.

The muscle underneath the surface of the foot was of a high density, and most of the muscle ran in the dorsal-ventral direction along the long axis of the foot. In the inner parts of the foot, the orientation of the muscle varied, such as in the anterior-posterior and right-left directions, and the density of muscle decreased. The middle part of the foot consists of the haemocoel with digestive ducts, digestive glands and gonad cells (Fig. 1J–L). The superficial muscle layer remained in the proximal side of the foot, and extended to the dorsal side and connected with the shell in the dorsal side (*em* in Fig. 1J). The posterior side of the wall connected with the anterior aortic valve (*em* in Fig. 1K), Keber's valve (Fig. 1D) and the dorsoventral muscle (Fig. 1L). Therefore, the anterior aorta, pedal artery, pedal sinus, intestine and stomach with digestive glands were inside the wall of the superficial layer of the muscle of the foot. We named this space the foot chamber. The position of the wall of the foot chamber was depicted by a red bold line in Fig. 1A. The visceral sinus, the heart, the kidney, the vascular system in the gill, and adductor muscles were outside of the wall.

### Cardiac cycle of the heart and motion of the foot

**Fig. 2.**
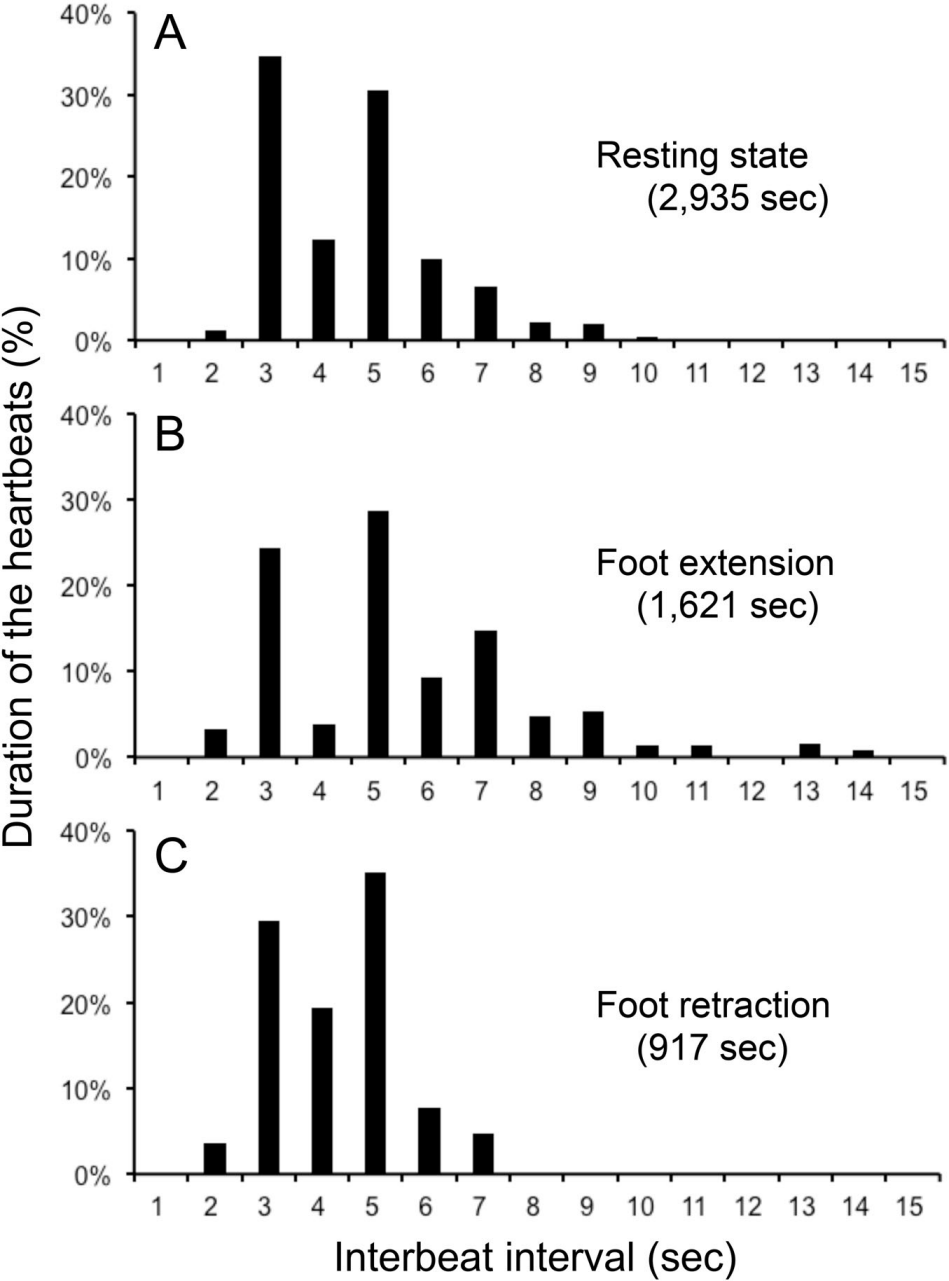
**Histogram of duration of the heartbeats by IBI during the resting state, extension and retraction of the foot.** The duration of the heartbeats was calculated by IBI x the number of beats. Duration was presented as the % of the total duration in each condition shown in parenthesis. Definitions of the three conditions are presented in the text. The number of mussels was seven, and 79 MRI sessions were observed at 20°C.

The interbeat interval (IBI) of the cardiac cycle was measured in 106 MRI sessions for 163 min in seven mussels at 20°C. Since the IBI showed a broad distribution from 2.5–15 s, heartbeats were separated into three conditions: resting state, extension of the foot and retraction of the foot. The term ‘foot extension’ refers to cases where we detected an increase in the volume of the foot and/or an extension of the foot outside of the shell in the MR images. The term ‘foot retraction’ refers to cases where we detected a decrease in the volume of the foot and/or retraction of the foot into the shell in the MR images. The term ‘resting state’ refers to cases where we could not detect motion of the foot in the MR images during the entire length of the single related MR session. The mean of the IBI remained at around 4–5 s for 6 h (Fig. S1). However, the extension and retraction of the foot was mostly observed in the initial 3 h. Therefore, in order to compare the IBI of the three conditions under the same timing, we analysed IBI using 79 MR sessions for 134 min in seven mussels observed within 3 h. The descriptive statistical parameters in the three conditions described above are shown in Table 1. The distributions shown for the three conditions showed high values for skewness, and were not normal distributions (*P*<0.05). The means and s.d. of the IBI were 4.61±1.34 s, 5.05±2.03 s and 4.39±1.07 s for the resting state, foot extension and foot retraction, respectively. The IBI value for the foot extension condition was significantly longer than those of the resting state and the foot retraction conditions (*P*<0.01). There was no difference between the IBI values for the resting state and the foot retraction conditions. Histograms showing the duration of the heartbeats determined by IBI in the three conditions were shown in Fig. 2. During the foot extension, the heartbeat of longer IBI (≧7 s) increased, compared with that of the foot retraction condition.

**Table 1.**
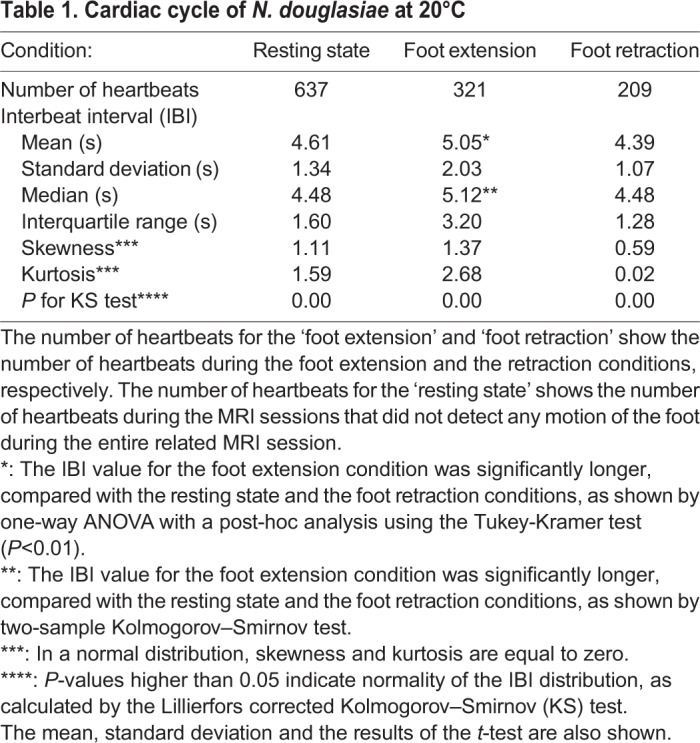
**Cardiac cycle of *N. douglasiae* at 20°C**

### Circulation in the resting state

**Fig. 3.**
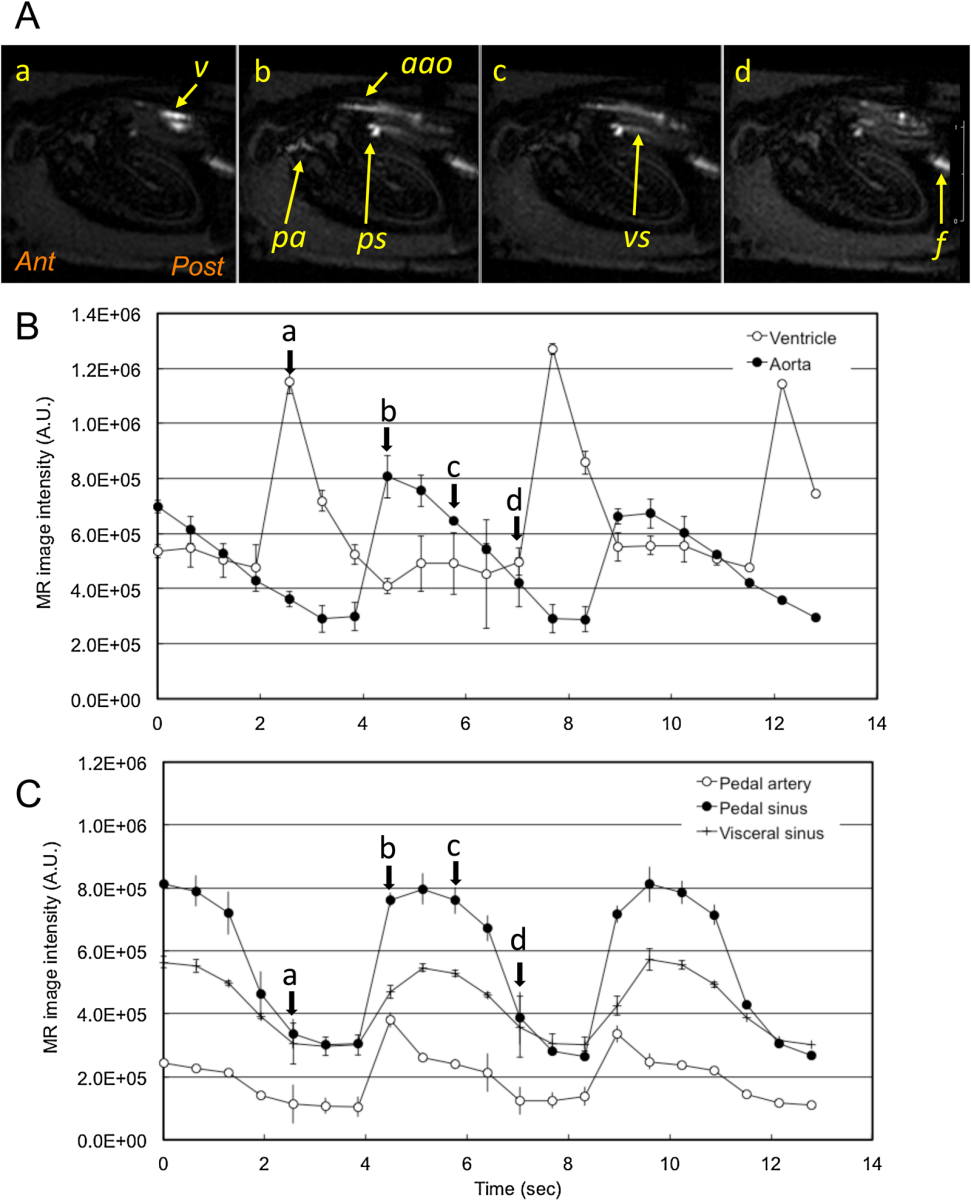

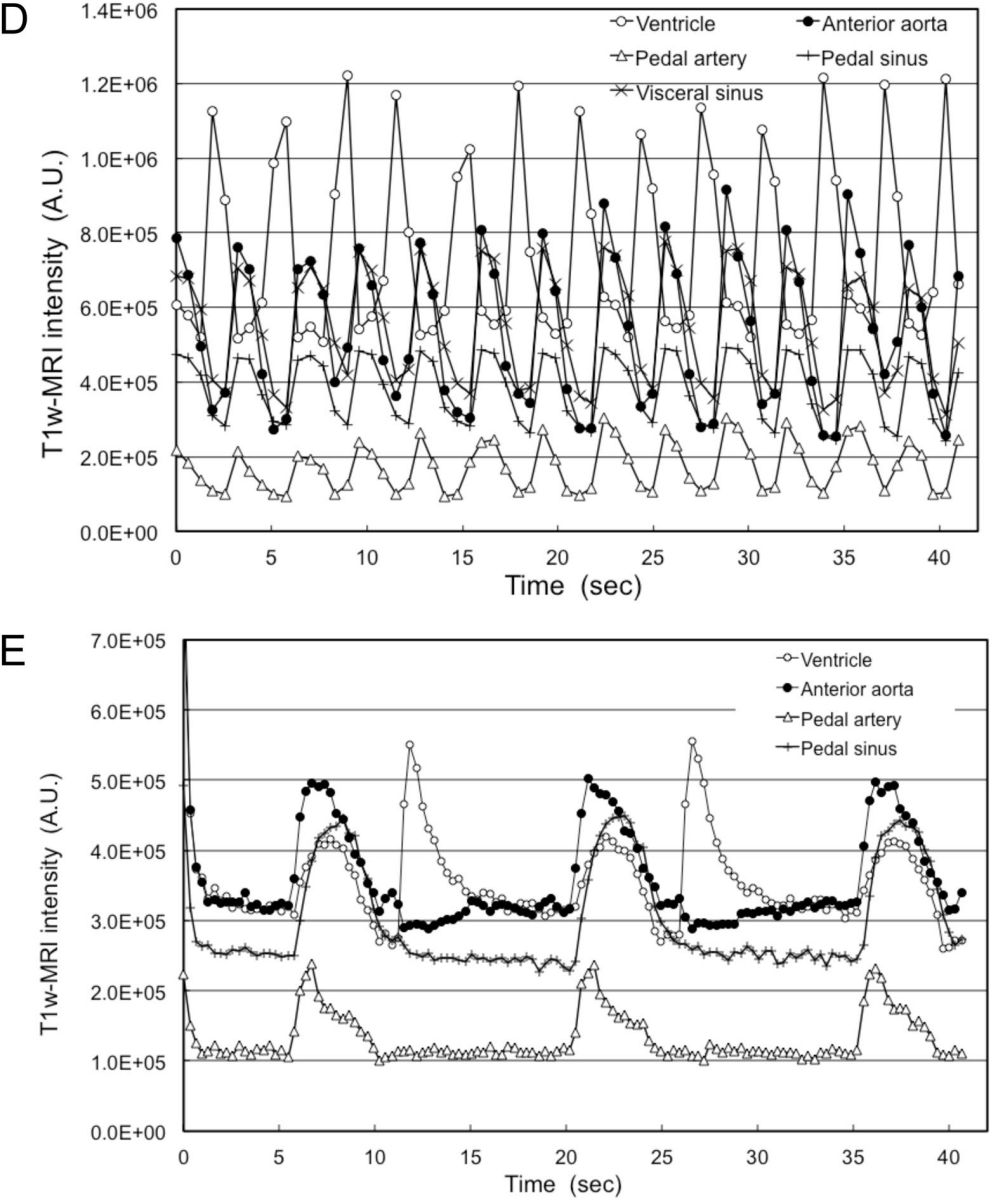
**Circulation during the resting state.** (A) Mid-longitudinal T_1w_-MR image during the resting state. (a) Diastolic phase of the ventricle. (b) Beginning of the systolic phase. (c) Middle of the systolic phase. (d) End of the systolic phase just before the diastolic phase. (B,C) Changes in image intensity of the ventricle, anterior aorta, pedal artery, pedal sinus and visceral sinus with an IBI of 4.9 s. Mean and s.d. of five pairs of heartbeats observed in an MRI session were plotted. Arrows labelled a to d show the timing of the T_1w_-MR images. Also refer to Movie 2. (D) This shows typical changes in the image intensity of the ventricle, anterior aorta, pedal artery, pedal sinus and visceral sinus with an IBI of 3.2 s. (E) Showing typical changes in image intensity of the ventricle, anterior aorta, pedal artery and pedal sinus with an IBI of 14.7 s observed at 9 h immersion in 12 ml water without aeration. The visceral sinus could not be detected in this image. Labelled features: *aao*, anterior aorta; *f*, water stream in the upper mantle cavity underneath the *PAM*; *pa*, pedal artery; *ps*, pedal sinus; *V*, ventricle; *vs*, visceral sinus.

The IBI during the resting state showed a wide variation (Fig. 2A). However, in each MRI measurement session, the IBI remained almost constant. A typical set of T_1w_-MR images and changes in image intensity were shown in Fig. 3 and Movie 2. The average IBI during the MRI session (81 s) was 4.9±0.40 s (mean±s.d., *n*=15). During the diastolic phase of the ventricle (Fig. 3Aa) the image intensity of the ventricle increased, which corresponded to the filling of the ventricle with haemolymph. No flow was detected in the anterior aorta, pedal artery, pedal sinus and visceral sinus. In the beginning of the systolic phase (Fig. 3Ab) the image intensity of the anterior aorta and the pedal artery increased instantaneously, corresponding to the increase in haemolymph flow. Then flow decreased gradually for 3 s. The pedal sinus returns from the right side of the foot (Fig. 1C); therefore, only the terminal part of the pedal sinus was detected in this longitudinal sagittal image (Fig. 3Ab). The image intensity of the pedal sinus and the visceral sinus reached a peak with a 1 s delay. The flow continued for 2 s (Fig. 3Ac), then decreased at the end of the systolic phase (Fig. 3Ad). In cases where the IBI was shorter, for example 3.2 s (Fig. 3D), the duration of the filling of the ventricle and the outflow of the anterior aorta became shorter. The duration of flow in the pedal and visceral sinuses was also shortened, and the timing of the peak flow almost synchronized with that of the anterior aorta. In cases where the IBI was longer, for example 14.7 s (Fig. 3E) observed under a hypoxic condition, the contraction time of ventricle became longer and flow in the pedal artery and the pedal sinus ceased completely until the next contraction of the ventricle. The filling of the ventricle occurred followed by ventricular contraction. Then the ventricle waited until the next contraction at the end diastolic position.

### Circulation during foot extension

**Fig. 4.**
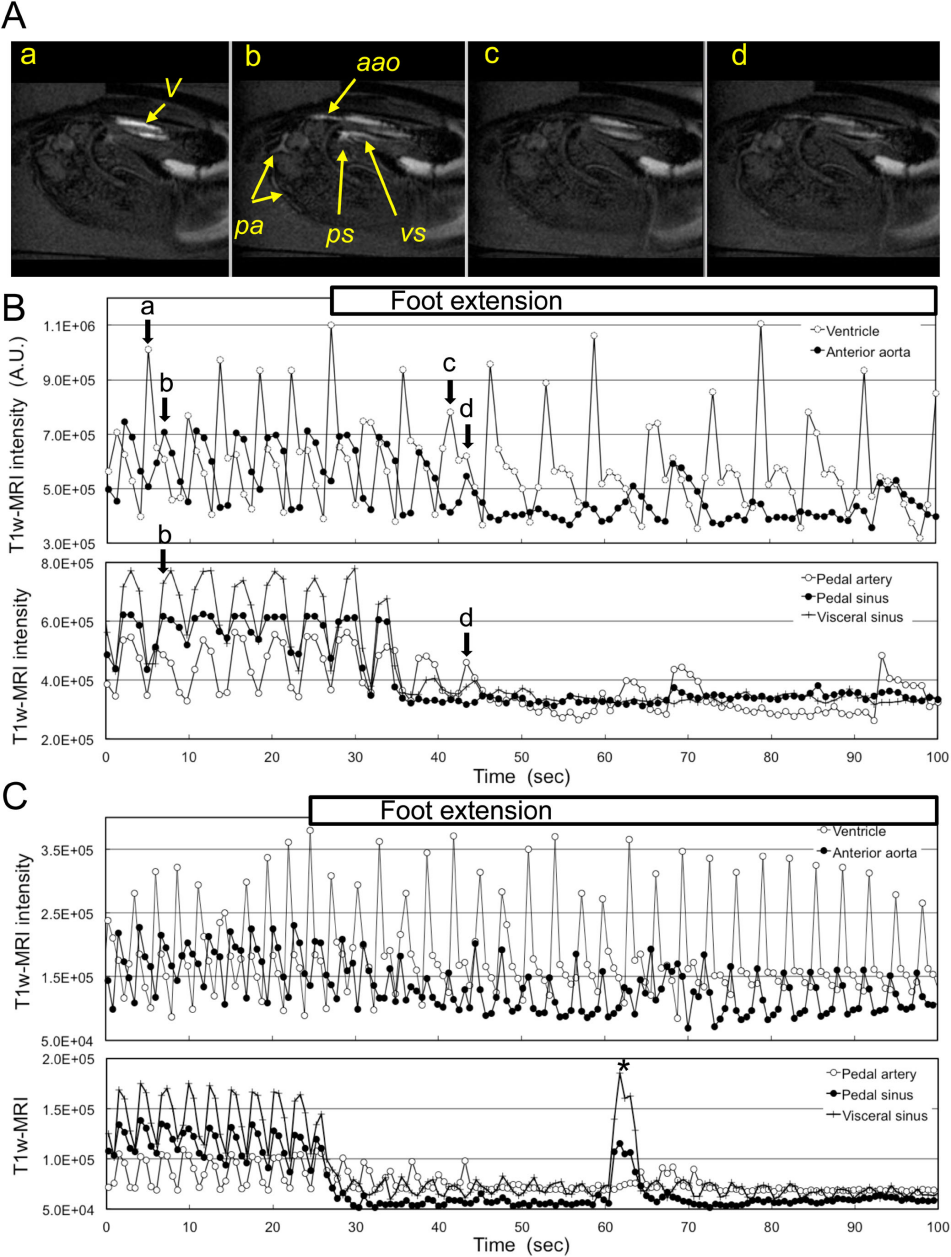
**Circulation during the foot extension.** (A) Mid-longitudinal T_1w_-MR image. a and b are images of the diastolic phase and systolic phase during the resting state, respectively. c and d are images of the diastolic phase and the systolic phase during foot extension, respectively. (B) Showing typical changes in the image intensity of the ventricle, anterior aorta, pedal artery, pedal sinus and visceral sinus. Labels a to d show the timing of the T_1w_-MR images. Extension of the foot started from around 27 s, and continued until the end of the MRI session. Arrows labelled a to d show the timing of the T_1w_-MR images. (C) Showing typical changes in image intensity of the ventricle, anterior aorta, pedal artery, pedal sinus and visceral sinus. The * indicates a spontaneous leakage of Keber's valve during the foot extension. Also refer to Movie 3. Labelled features: *aao*, anterior aorta; *pa*, pedal artery; *ps*, pedal sinus; *V*, ventricle; *vs*, visceral sinus.

During foot extension the circulation of the mussel changed dramatically. Typical cases of a persistent extension of the foot for more than 60 s are shown in Fig. 4 and Movie 3. The mussel started to extend the foot from 27 s (Fig. 4B). The flow in the visceral sinus and the pedal sinus stopped after two heartbeats. The flow in the pedal artery also decreased gradually and almost stopped after four heartbeats (Fig. 4Ac,d,B). Flow through the anterior aorta also decreased gradually, but tended to be maintained (Fig. 4B). Meanwhile, the ventricle continued to contract during the foot extension, even though the IBI increased to 6.3±1.0 s (mean±s.d., *n*=15) from that of the resting state (4.3±0.5 s, *n*=10, *P*<0.01). Changes in the flow during 27 foot extension cases are summarized in Table 2. Ventricular contraction did not stop during the foot extension, but the IBI increased in three-quarters of the foot extension cases. In almost 90% of these cases, the flow in the visceral sinus and the pedal sinus stopped within three heartbeats. Meanwhile, flow in the pedal artery continued for longer than three heartbeats in 60% of these cases. During a persistent extension of the foot, the peak flow of the anterior aorta moved from after the ventricular filling to before the ventricular filling (Fig. 4C). This delay is probably caused by an increase of output resistance due to the increase in the pressure in the foot haemocoel. Spontaneous increases in the flow in the pedal sinus and the visceral sinus were also observed in some cases (* in Fig. 4C).

**Table 2.**
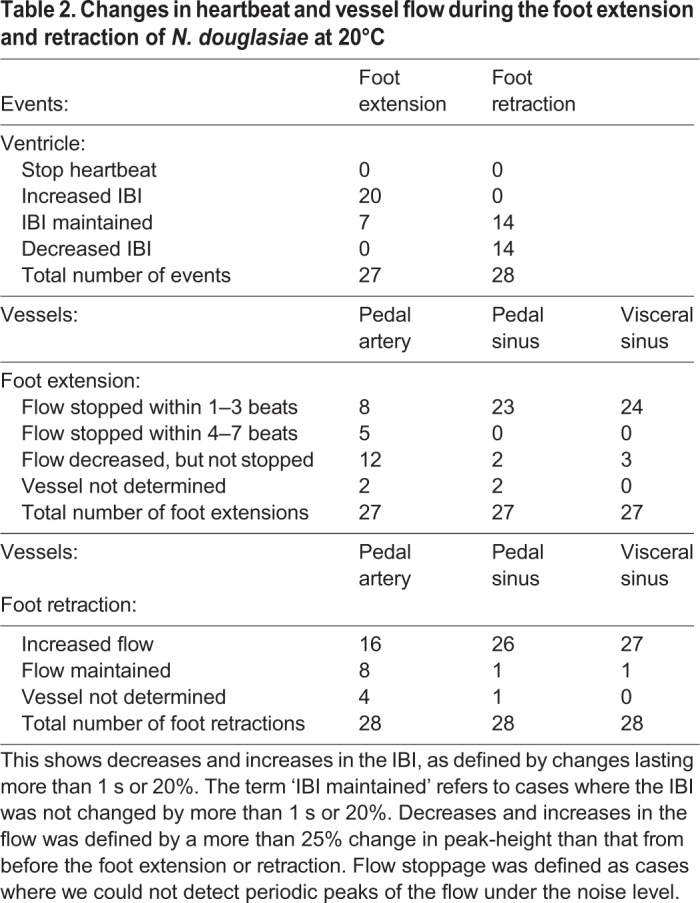
**Changes in heartbeat and vessel flow during the foot extension and retraction of *N. douglasiae* at 20°C**

### Circulation during foot retraction

**Fig. 5.**
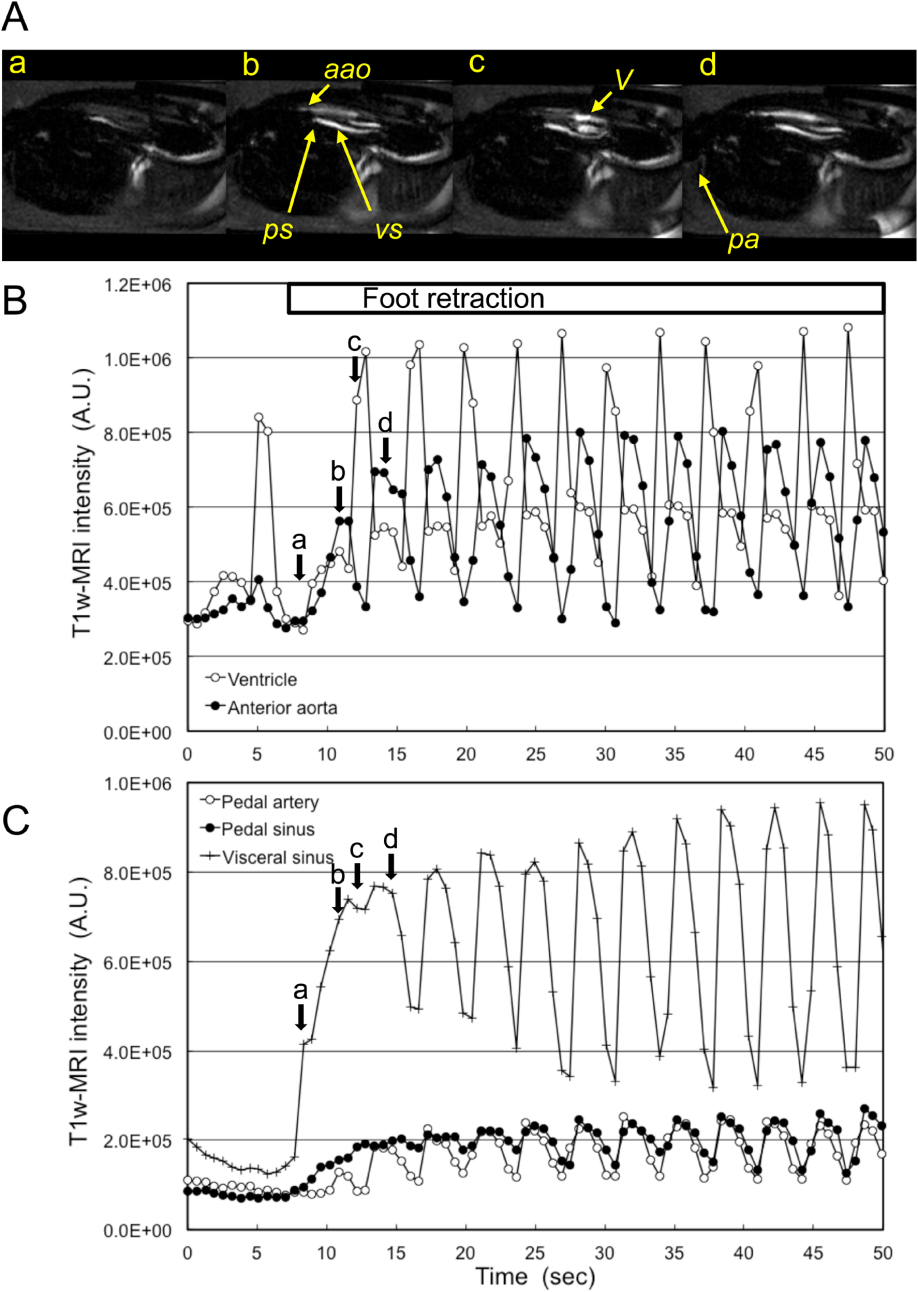
**Circulation during the foot retraction.** (A) Mid-longitudinal T_1w_-MR image at the foot retraction. (a) Beginning of the foot retraction. (b) Systolic phase. (c) Diastolic phase. (d) Systolic phase. (B,C) Showing typical changes in T_1w_-MR image intensity of the ventricle, anterior aorta, pedal artery, pedal sinus and visceral sinus. Retraction of the foot started from around 8 s, and continued until the end of the MRI session. Arrows labelled a to d show the timing of the T_1w_-MR images. Also refer to Movie 4. Labelled features: *aao*, anterior aorta; *pa*, pedal artery; *ps*, pedal sinus; *V*, ventricle; *vs*, visceral sinus.

Changes in the flow during 28 cases of foot retraction are summarized in Table 2. Ventricular contraction did not stop during the foot retraction. The IBI decreased 50% in the foot retraction cases, but the IBI did not show any changes in the rest of the cases. In more than 90% of these cases, the flow in the pedal sinus and visceral sinus was increased during the retraction. A typical foot retraction case is shown in Fig. 5 and Movie 4. Detection of the exact timing of start of the foot retraction by MRI is difficult, compared with that of foot extension, because the end of the foot was usually outside of the FOV of the MR image. However, the increase in the flow in the pedal sinus and the visceral sinus might be the initial event that occurs in the foot retraction process. The initial flow in the pedal sinus and visceral sinus was continuous, but then turned into a pulsated flow after 10–20 s of the foot retraction. Meanwhile, flow in the anterior aorta and the pedal artery was always pulsated while the heart was beating.

## DISCUSSION

### The function of Keber's valve and the dorsoventral muscle

This is the first study to visualize dynamic changes in the haemolymph flow through Keber's valve, and also the first to confirm the flow direction from the pedal sinus to the visceral sinus. Keber's valve was first reported by Keber (1851) as a ‘zweilippingen Oeffnung’ (double-lip opening) between the pedal sinus and the visceral sinus, and Keber suggested that it works as a venous lock in combination with the ‘kleine längliche Muskel’ (dorsoventral muscle). Compared with Keber's valve in *Anodonta cygnea* (Fig. 7 in Willem and Minne, 1898), *Anodonta cellensis* (Figs 26 and 27 in Schwanecke, 1913) and *A. anatina* (Brand, 1972), Keber's valve in the *N. douglasiae* is similar in shape to a sphincter muscle. The flow in the pedal and visceral sinuses were usually synchronized with each other, but in some cases, such as the initial flow in the foot retraction condition (Fig. 5B) and the flow in the pedal sinus, the flow was continuous, while the flow in the visceral sinus was a pulsated one. This phenomenon also supports the concept that Keber's valve is the major factor controlling the venous return from the foot. We could not visualise the dorsoventral muscle *in vivo* MRI. Thus, we could not detect that effect on the flow separately from that of Keber's valve. Histologically, the dorsoventral muscle is a thin, flat, trapeziform muscle (Movie 1). The shape is different from the thin elongated muscle seen in *A.* *cygnea*, *A.* *cellensis* and *A.* *anatina*. The dorsal end of the dorsoventral muscle connected with the dorsal lip of Keber's valve and the ventral parts were connected with the muscle of the foot chamber. Therefore, even though the wall of the visceral sinus is a thin renal epithelia wall, contraction of the dorsoventral muscle might be useful to increase the flow resistance of the visceral sinus, which would function to assist Keber's valve.

### Circulation at resting state

As shown in Fig. 3, flow in the pedal sinus and the visceral sinus was pulsated and it was synchronized with the ventricular contraction with a minimum delay, even with a wide range of IBI. In the open circulatory system we are studying here, it is unlikely that the pulsated flow of the pedal artery reaches the pedal sinus, since the haemolymph has to have a way out into the interstitial space from the vessel, where the pressure has to decrease due to the large volume of the interstitial space. Indeed, Brand reported a significant decrease in the pressure pulse in the pedal haemocoel in *A.* *anatine* (Fig. 7 in Brand, 1972). Thus, it has been expected that sinus flow is continuous. As mentioned in Fig. 1J–L, the superficial muscle of the foot extended to the dorsal part of the shell, and covered not only the foot but also the vessels and digestive organs in the anterior part of the mussel. Therefore, the superficial muscle and the dorsal part of the shell consists of the foot chamber with an inlet (the anterior aorta) and an outlet (Keber's valve) (Fig. 1A). If the tension of the superficial muscle is high enough to maintain a low compliance of the wall of the foot chamber, the pressure pulse applied by the anterior aorta could transfer to Keber's valve with a minimum decrease and delay. Thus, it might be true that there is a low compliance in the foot chamber. As shown in Fig. 3D and E, the sinus flow was pulsated even with a short IBI of around 3 s, and the sinus flow followed changes in the flow of the anterior aorta even with a long IBI at around 15 s. If the compliance is not low enough, the sinus flow should become continuous at a short IBI, and it should also decrease due to the increase in the foot volume during the long-lasting pulsated inflow from the anterior aorta. The low compliance of the wall of the foot chamber could maintain venous return from the foot via Keber's valve, and need not assist the negative pressure in the auricle caused by the ventricular contraction (constant-volume hypothesis) (Ramsay, 1952; Krijgsman and Divaris, 1955; Seo et al., 2014a).

The discussion above supposes a passive movement for Keber's valve. At present, we have no evidence that could deny active control of Keber's valve during the resting state. Further studies are necessary to clarify the control of Keber's valve in the resting state of the mussel.

### Circulation during foot extension and retraction

During the foot extension, in almost 90% of the cases, flow in the pedal and visceral sinuses stopped within three heartbeats. Meanwhile, flow in the pedal artery continued for longer than three heartbeats in 60% of the cases. In *A.* *anatine*, the pressure of the haemocoel increased gradually followed by an increase of the systolic ventricular pressure up to 50 mm H_2_O during the initial extension and probing of the foot into the sand (Brand, 1972, 1976). Therefore, it was expected that Keber's valve would be closed at the beginning of the foot extension. However, as far as we have observed, closure of Keber's valve was delayed at the start of the extension. In the beginning of the foot extension, the muscle of the foot chamber might relax and the compliance of the foot would increase, and as a result the foot would start to extend. In order to extend further, Keber's valve is actively closed so that the pressure in the foot haemocoel is increased by the inflow via the pedal artery. The increase of the pressure in the foot haemocoel could reduce the flow in the pedal artery (Fig. 4) and also increase the systolic pressure of the ventricle (Brand, 1972, 1976). The control mechanism of Keber's valve is unknown, but it might be expected that the nervous control system is involved. A short leakage of the flow seen in the pedal sinus and the visceral sinus during persistent extension of the foot (* in Fig. 4C) also suggests the presence of an active control system for Keber's valve.

The IBI increased during the foot extension, compared with the resting condition (Fig. 2, Table 1). When Keber's valve closed, venous return to the heart was decreased. Therefore it takes longer to fill the ventricle of the heart. This is one reason for the increase in the IBI suggested by Brand (1976) and da Silva Cândido and Brazil Romero (2006). The *N. douglasiae* has a posterior aorta that supplies haemolymph to the posterior part and the mantle of the mussel, and the haemolymph will return to the auricles via the gill. During the foot extension, the output resistance of the anterior aorta increased. But it may not cause a significant increase in the flow of the posterior aorta, because flow resistance of the posterior aorta might be 80 times of that of the anterior aorta, judging from the ratio of the cross-sectional area of the aortae. The vascular system of the gill and the heart are outside of the foot chamber (Fig. 1J-L); therefore, the circulation of the gill is probably not affected by the extension of the foot.

The retraction of the foot may start from the opening of Keber's valve since the flow in the pedal and visceral sinuses was increased during the retraction in more than 90% of the cases we studied. The initial flow was increased by the efflux of haemolymph due to the decrease in foot volume and due to the contraction of the retractor muscles. Therefore, the initial flow of the pedal sinus and visceral sinus was continuous, which then turned into pulsated flow thereafter. The IBI decreased, compared with the foot extension (Fig. 2, Table 1). When Keber's valve was open, the venous return was increased; thus, it took a shorter time to fill the ventricle of the heart. Therefore, any increase or decrease in the IBI during foot extension and retraction is likely to reflect a decrease and increase in the venous return, respectively, and it is not likely to be controlled by the activity of the mussel.

In conclusion, at rest a low compliance of the wall of the foot chamber could function to maintain the venous return via Keber's valve, so that the volume of the foot was constant. Extension of the foot is initiated by relaxation of the foot muscle. Then Keber's valve actively shuts the outflow, so that the foot extends further due to an increase in the volume of the haemolymph in the foot haemocoel. Retraction of the foot is initiated by the opening of Keber's valve, followed by an increase in the outflow of the haemolymph with a combination of a contraction of the retractor muscle. Based on these observations, the conventional model of extension and retraction of the foot can now be revised. Keber's valve and the foot chamber are essential, not only for extension and retraction of the foot, but also for circulation at rest. We admit that in this experiment the mussel was not placed in the sand, but was positioned in a plastic tube using a piece of elastic strip which was inserted at the hinge position of the shell. So the mussel could extend the foot freely in water until the foot touched the wall of the plastic tube. Therefore, we could observe the initial phase of the foot extension, but we are afraid that the initial extension of the foot observed in this experiment could not be observed in real sand due to resistance by the sand. In order to study the burrowing process, we are now planning a study designed to observe the motion of the foot in sand.

## MATERIALS AND METHODS

### Experimental mussels

*Nodularia douglasiae* (Gray, 1833) were supplied by Sato Craft (Okayama, Japan). These mussels were collected from a river in Seki, Okayama in June of 2016. The species of mussel was identified by sequencing of the mitochondrial cytochrome c oxidase subunit I gene. DNA was extracted from the mantle using the DNeasy Blood and Tissue Kit (Qiagen, Hilden, Germany). The extracted DNA was used as the template for PCR reactions. PCR amplifications of the mitochondrial COI gene fragments were carried out using the universal primers LCO1490 and HCO2198 (Folmer et al., 1994). Purified PCR products were sequenced bi-directionally using ABI 3130xl Genetic Analyzer (Applied Biosystems Japan Ltd., Tokyo, Japan). The sequence was analysed using the gapped-BLAST search algorithm to estimate the degree of similarity to other relative sequences deposited in GenBank. As a result, the sequence was coincident with *N. douglasiae* (GenBank accession number KT984766). After collection, the mussels were immersed in water and then transported to the laboratory by a refrigerated transport service, maintained at 10°C, within a time period of 16 h (Cool Ta-Q-BIN, Yamato Transport Co., Ltd., Tokyo, Japan). At the laboratory, the mussels were housed in aerated water (4 l) with glass beads (0.3–1.0 mm diameter, 5 cm depth) in a 6 l bath at room temperature (20–25°C). The mussels were fed with *Chaetoceros calcitrans* (WDB Environmental & Biological Research Institute, Tokushima, Japan), and 2.5×10^6^ cells/l water in the bath was applied at intervals of 3–4 days. The experiments were conducted 1–5 weeks after sampling. A total of seven mussels were used in this study. The length, height and width of mussels were 36.4±2.3 mm, 20.5±0.7 mm, 14.5±1.0 mm (mean and s.d.), respectively. Motion of the foot, flow of haemolymph and heartbeats were detected by MRI in seven live mussels. After *in vivo* MRI experiments, four out of the seven mussels were fixed with 4% paraformaldehyde (PFA) in order to obtain high spatial resolution MRI, and one out of the four fixed mussels was used for histological sections. All of the animal experiments conducted in this study were carried out under the rules and regulations of the ‘Guiding Principles for the Care and Use of Animals,’ as approved by the Council of the Physiological Society of Japan.

### Magnetic resonance imaging

The ^1^H magnetic resonance (MR) images were obtained with ParaVison operating software (version 5.1), using a 7 T microimaging system (AVANCE III, Bruker Biospin, Ettlingen, Germany) equipped with an active shielded gradient (micro2.5) and a 25-mm ^1^H birdcage radiofrequency coil. The mussels were placed in a plastic tube (inner diameter of 22.5 mm), and each mussel was positioned in place using a piece of elastic silicone strip which was inserted at the hinge position of the shell. The mussels were immersed in 12 ml of water without aeration, and the temperature was kept at 20°C by variable temperature control units (ECU-20 and BVT-2000, Bruker Biospin, Baden-Württemberg, Germany). The stability of the temperature was monitored by a fluorescence thermometer (AMOS FX-8000-210, Anritsu Meter, Tokyo, Japan) before, during and after the MRI session (Seo et al., 2014a).

### Motion of the heart and foot

Motion of the heart and foot were imaged using T_1_-weighted gradient-echo imaging (T_1w_-MRI) (Bock et al., 2001; Seo et al., 2014b, 2016). The typical sagittal imaging parameters used for the *in vivo* T_1w_-MRI were as follows: 46.08×23.04 mm field of view (FOV) with a 128×64 data matrix and a slice thickness of 1 mm or 2 mm, 10 ms relaxation delay (TR), 3.5 ms echo-time (TE), 22.5° or 16° flip angle, and 1 accumulation. A transverse image was obtained by 23.04×23.04 mm FOV with a slice thickness of 1 mm or 2 mm. Each MRI measurement session consisted of 128 or 256 images obtained by intervals of 0.64 s. For higher heart rates, images were obtained by intervals of 0.32 s using a 64×32 data matrix. For higher image resolution, images obtained by intervals of 0.96 s using a 256×128 data matrix, but were not used for analysis of distribution of IBI. In order to prevent heating due to radio-frequency pulse, the MRI sessions were repeated for at least a 15-min interval (Seo et al., 2016). The measurements were finished within 6 h, except for one MRI session that was observed at 9 h, and all of the mussels survived after the MRI measurements.

Heartbeat was shown by the IBI that was calculated from the peak-to-peak interval of T_1w_-MR image intensity of the ventricle of the heart. Normality of the distribution of IBI was tested by the Lillierfors corrected Kolmogorov–Smirnov test employing IBM SPSS Statistics software (V25, IBM, New York, USA). *P*-values higher than 0.05 were taken to indicate normality. Significant differences between the IBI was tested using the two-sample Kolmogorov–Smirnov test, and/or using one-way ANOVA with a post-hoc analysis using the Tukey-Kramer test employing IBM SPSS Statistics software (V25, IBM, New York, USA). *P*-values less than 0.01 were regarded as significant.

### Haemolymph flow

The haemolymph flow in the vessels was imaged by the inflow effect of T_1w_-MRI. The image intensity increased when the flow velocity increased (Bock et al., 2001; Seo et al., 2014b). The direction of flow was measured by phase-contrast gradient-echo sequences (PC-MRI) (Lotz et al., 2002; Seo et al., 2014b), using a transverse slice with a pixel resolution of 90×90 µm and a slice thickness of 1 mm with a combination of TR/TE/flip angle=100 ms/10 ms/45°. Eight pairs of velocity encoding gradients were used, with a strength corresponding to a velocity from −11.25 to 15 mm s^−1^ with a 3.75 mm s^−1^ step, and a total image acquisition time of 8 min 42 s. The lower limit of detection of flow was ±3.75 mm s^−1^.

### Anatomical structure

Anatomical information for the PFA fixed mussel was obtained by 3D MRI. The imaging parameters used for 3D T_1_-weighted gradient-echo imaging (3D T_1w_-MRI) included a voxel size of 60×60×60 µm, and a combination of TR/TE/flip angle=50 ms/3.75 ms/45°. For the histology, the PFA fixed mussel was embedded in paraffin wax after dehydration. The paraffin sections were prepared using a slice thickness of 10 µm. The sections were stained by HE staining. Images were detected by a microscope (BZ-X710, Keyence, Osaka, Japan) with an image-stitching mode. Photoshop Elements (10.0, Adobe, San Jose, USA) was used to select the ROI and to adjust the position of 22 images around Keber's valve, obtained at 50 µm intervals.

## Supplementary Material

Supplementary information
