# Supplementary material for: Roles of Keber's valve and foot chamber for foot manipulation in the mussel Nodularia douglasiae
Source: Biol Open. 2018 Dec 10;8(1):bio039859. doi: 10.1242/bio.039859 (PMC6361212; doi:10.1242/bio.039859)
Supplement: Supplementary information [file biolopen-8-039859-s1.pdf]

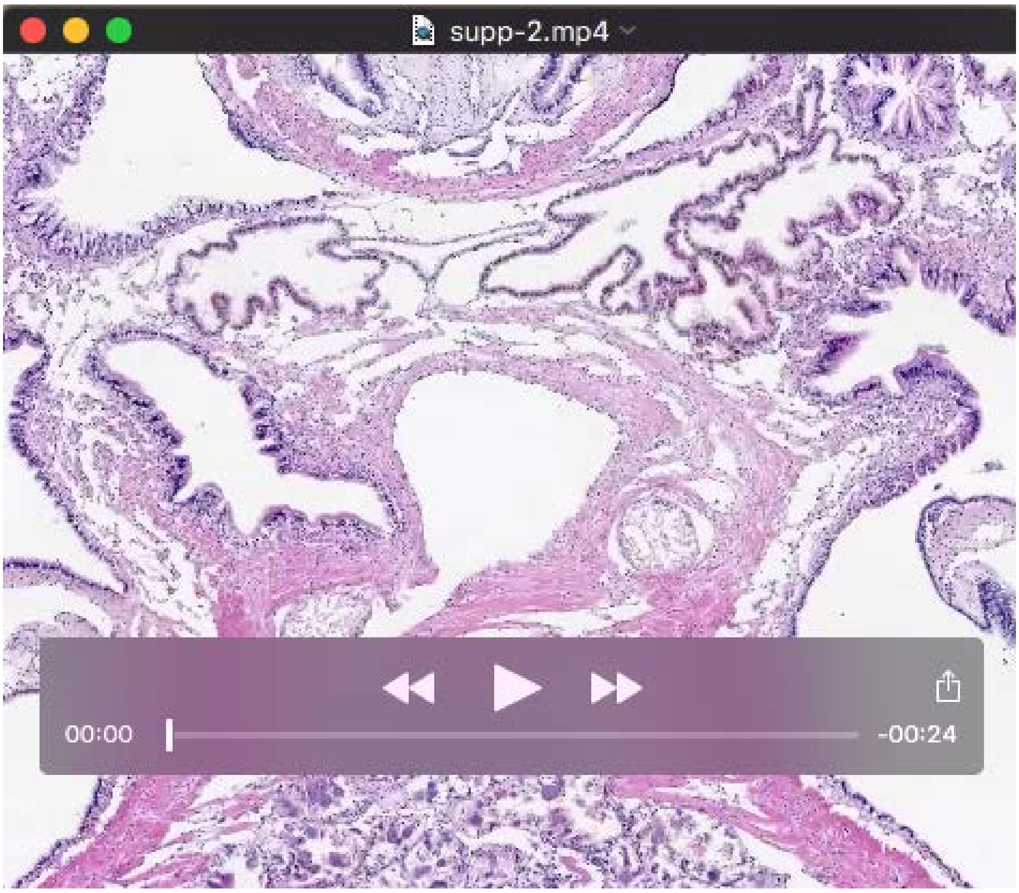

Movie 1. Transverse HE images of the PFA fixed *Nodularia douglasiae* from the pedal sinus, Keber's valve to the posterior end of the dorsoventral muscle. Twenty two images were obtained at 50 μm interval.

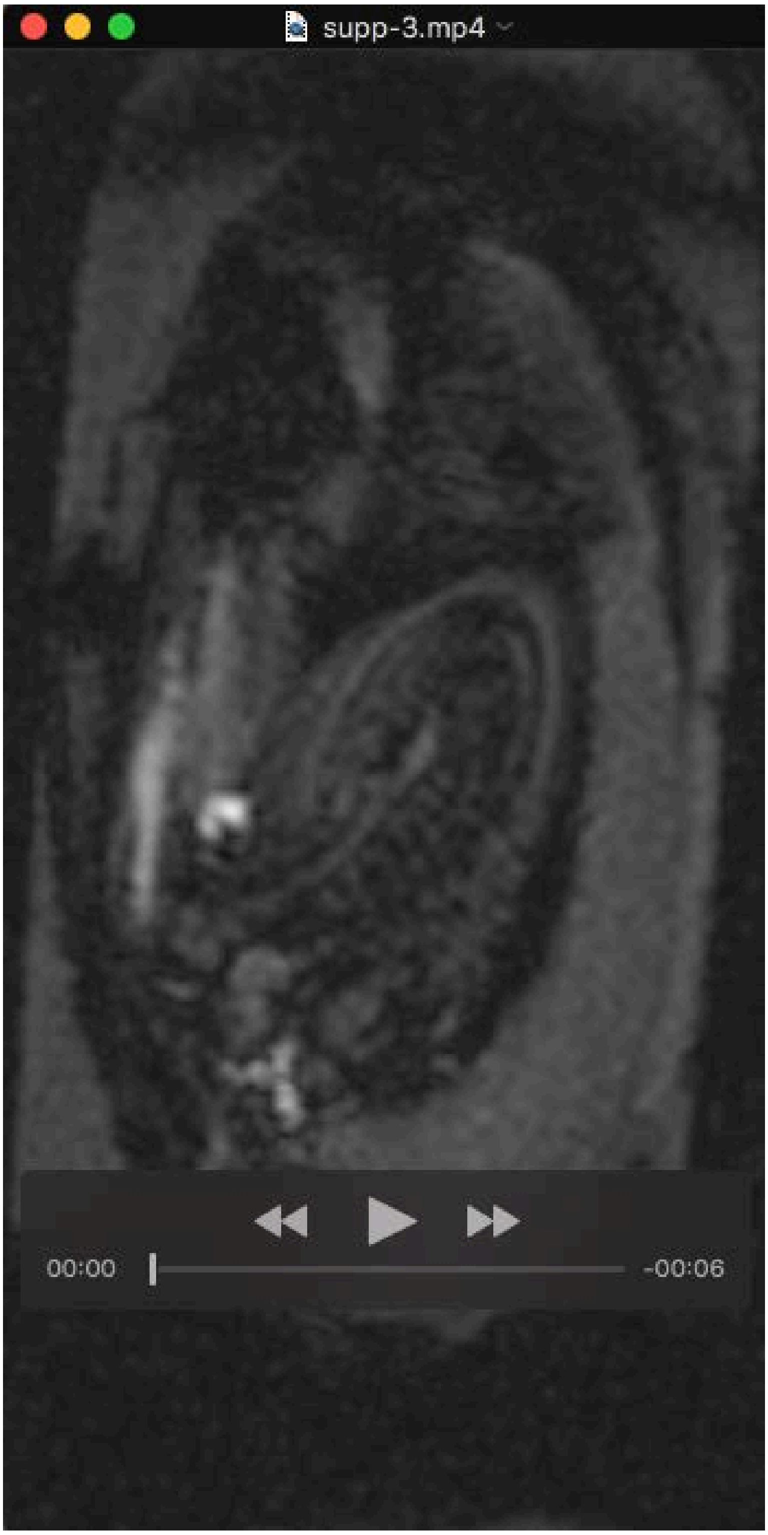

Movie 2. Circulation during the resting state. Mid-longitudinal T_{1w}-MR image during resting state obtained every 0.64 s. From original images shown in Fig. 3, 64 images are replayed at 10 flames per second.

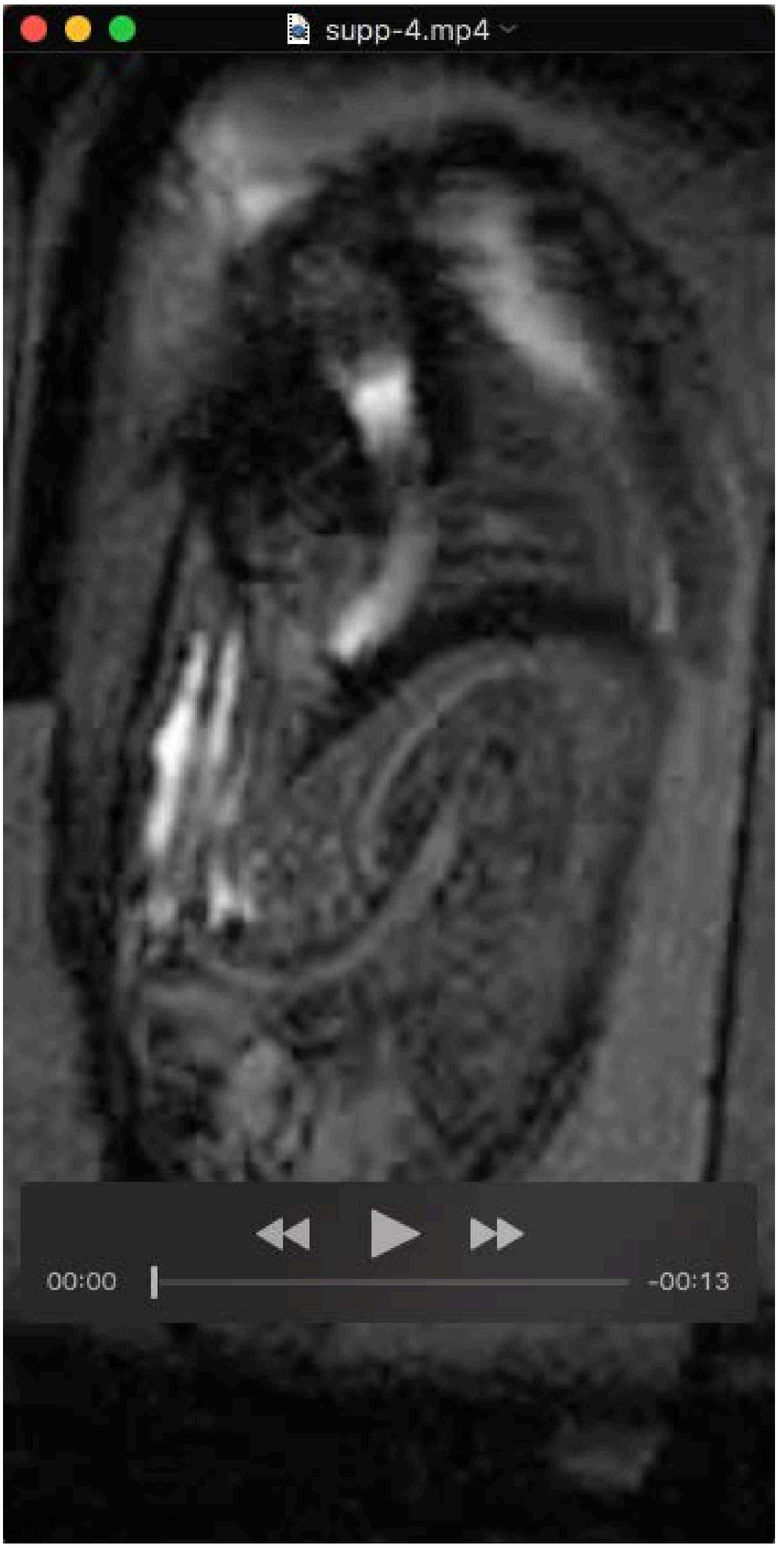

Movie 3. Circulation during the foot extension. Mid-longitudinal T_{1w}-MR image from the resting state to the foot extension obtained every 0.64 s. From images shown in Fig. 4C, 128 images from 0 s to 81 s are replayed at 10 flames per second.

Biology Open • Supplementary information

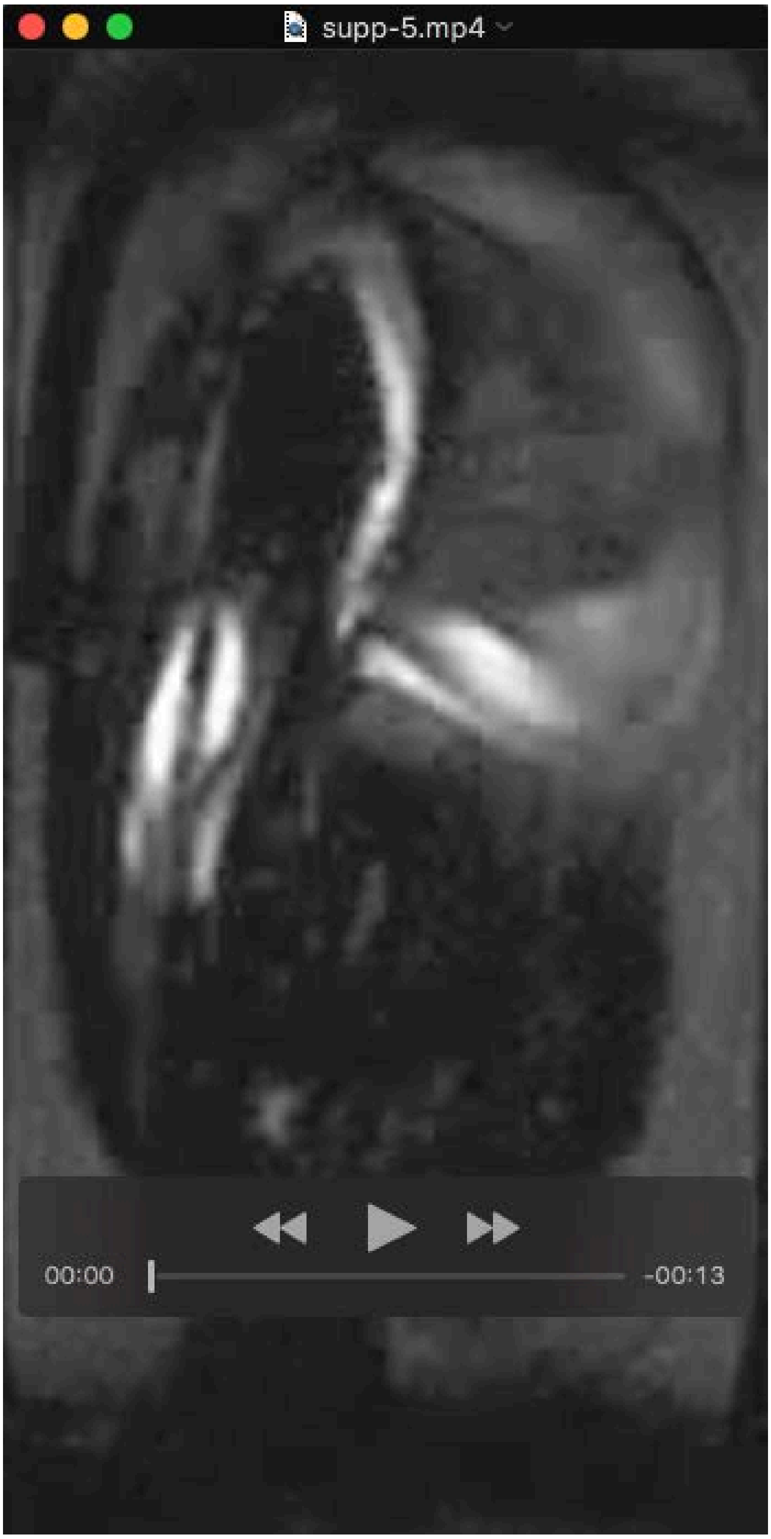

Movie 4. Circulation during the foot retraction. Mid-longitudinal T_{1w}-MR image from the resting state to the foot extension obtained every 0.64 s. From images shown in Fig. 5C, 128 images from -10 s to 71 s are replayed at 10 flames per second.

Fig. S1. Effects of hypoxic condition on the interbeat interval of *Nodularia douglasiae* at 20°C. Means and s.d. of the interbeat interval (IBI) from 4 clams were calculated from the MR session during 0.25-1.5 h, 1.5-3.0 h, 3.0-4.5 h and 4.5-6.0 h after setting the sample tube with 12 mL water without aeration. Statistical significant difference was detected between means of IBI by ANOVA (P < 0.01). The post-hoc analysis also showed significant differences, except for the pair of clams at 1.5-3.0 h and 4.5-6.0 h..

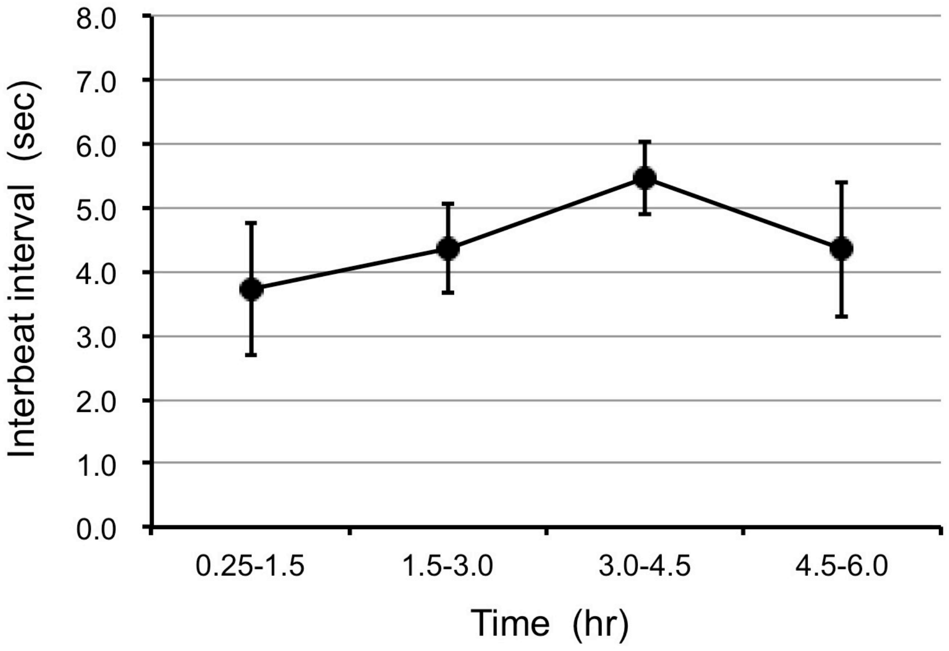

Biology Open • Supplementary information
